# Towards digital health equity - a qualitative study of the challenges experienced by vulnerable groups in using digital health services in the COVID-19 era

**DOI:** 10.1186/s12913-022-07584-4

**Published:** 2022-02-12

**Authors:** Anu-Marja Kaihlanen, Lotta Virtanen, Ulla Buchert, Nuriiar Safarov, Paula Valkonen, Laura Hietapakka, Iiris Hörhammer, Sari Kujala, Anne Kouvonen, Tarja Heponiemi

**Affiliations:** 1 Finnish Institute for Health and Welfare, P.O. Box 30, FI-00271 Helsinki, Finland; 2 Faculty of Social Sciences, University of Helsinki, PO Box 54, 00014 Helsinki, Finland; 3 Department of Computer Science, Aalto University, PO Box 15400, FI-00076 Aalto, Helsinki Finland; 4 Department of Industrial Engineering and Management, Aalto University, P.O. Box 11000, FI-00076 Aalto, Helsinki Finland; 5 Centre for Public Health, Queen’s University Belfast, Institute of Clinical Sciences, Royal Victoria Hospital, Belfast, BT12 6BA UK

**Keywords:** Digital health services, Vulnerable groups, Digital health equity, Digital determinants of health, COVID-19

## Abstract

**Background:**

The COVID-19 pandemic has given an unprecedented boost to already increased digital health services, which can place many vulnerable groups at risk of digital exclusion. To improve the likelihood of achieving digital health equity, it is necessary to identify and address the elements that may prevent vulnerable groups from benefiting from digital health services. This study examined the challenges experienced by vulnerable groups in using digital health services during the COVID-19 pandemic.

**Methods:**

Qualitative descriptive design was utilized. Semi-structured interviews were conducted between October 2020 and May 2021. The participants (*N* = 74) were older adults, migrants, mental health service users, high users of health services, and the unemployed. Qualitative content analysis with both inductive and deductive approach was used to analyze the data. Challenges related to the use of digital health services were interpreted through digital determinants of health from the Digital Health Equity Framework.

**Results:**

For most of the participants the access to digital health services was hampered by insufficient digital, and / or local language skills. The lack of support and training, poor health, as well as the lack of strong e-identification or suitable devices also prevented the access. Digital services were not perceived to be applicable for all situations or capable of replacing face-to-face services due to the poor communication in the digital environment. Fears and the lack of trust regarding digital platforms were expressed as well as concerns related to the security of the services. Contact with a health care professional was also considered less personal and more prone to misunderstandings in the digital environment than in face-to-face services. Finally, digital alternatives were not always available as desired by participants, or participants were unaware of existing digital services and their value.

**Conclusion:**

Several development needs in the implementation of digital health services were identified that could improve equal access to and benefits gained from digital services in the future. While digital health services are increasing, traditional face-to-face services will still need to be offered alongside the digital ones to ensure equal access to services.

**Supplementary Information:**

The online version contains supplementary material available at 10.1186/s12913-022-07584-4.

## Introduction

The COVID-19 pandemic has placed health care systems globally in an unprecedented position. The sudden spread of COVID-19 cases have required urgent actions and extension of services to digital form to maintain health care operations and reduce face-to-face encounters [1, 2]. The variety and the number of digital health services has increased significantly over the past decade already before the pandemic, but it has been argued that the COVID-19 crisis will revolutionize the delivery of health services through digital technology [3, 4].

Although the purpose of digital health services is to enable the availability and continuity of services during the pandemic, everyone does not have an equal opportunity to benefit from digitalization. The rapid digitalization of health services has posed a considerable risk of increasing digital inequality, which in turn may cause significant disadvantages such as an increased risk of health deterioration, exposure to COVID-19 if the necessary services cannot be obtained remotely, and social isolation, especially for those who already are in a vulnerable position [2, 5–7]. Concerns have been raised about whether particularly those, who may not have equal access, ability or resources to use digital services have received the health services they need [5, 8, 9].

The aim of this qualitative study was to examine the challenges experienced by vulnerable groups in using digital health services during the COVID-19 pandemic. In this study, digital health services refer to electronic transactions related to health care, such as remote visits with professionals via video call, chat service or phone call, electronic health records, electronic symptom assessments, websites providing health-related information, health applications, or appointment booking system.

### Digital health equity

Facilitating appropriate use of digital technologies without leaving anyone behind, thus ensuring digital health equity is one of the guiding principles in the WHO global strategy on digital health 2020-2025 [10]. Digital health equity can be defined as an equal opportunity for individuals to benefit from the knowledge and practices related to the development and use of digital technologies to improve health [10, 11]. Inequity in society is reflected in digital health equity, thus, those at risk of social exclusion (ie, those at a disadvantage due to, for example, unemployment, low education, and a weak economic situation) are also at greater risk of being excluded from digital health services [12, 13].

The realization of digital health equity is closely linked to realization of “digital determinants of health” [10], which, in turn, reflects the socio-economic and socio-cultural context of individuals and the intermediate health factors [11]. The Digital Health Equity Framework (DHEF) by Crawford and Serhal [11] defines the digital determinants of health as (1) individuals access to digital resources, (2) use of these resources for health seeking, (3) digital health literacy, (4) beliefs about the potential help or harm of digital health care, (5) values and cultural preferences regarding the use of digital resources, and (6) integration of digital resources into community and health infrastructure. In order to improve the likelihood of achieving digital health equity, there is a need to identify and address potential gaps in digital determinants, in particular from the perspective of those who are at risk of being excluded from digital services.

### Vulnerable groups as digital health service users

Previous literature has highlighted the need for public services to address the needs of vulnerable groups, those who are disadvantaged by health, economic, cultural or social conditions [14]. The key vulnerable groups include older people, migrants, mental health service users, high users of health services and the unemployed.

*Older adults* are the largest individual group that faces challenges in using digital health services [15–17]. Inexperience in the use of technology, poor motivation, financial difficulties, and insufficient technical skills have been shown to hinder older peoples’ opportunities to benefit from digital health services. In addition, poor health, cognitive decline, the lack of appropriate devices or internet access, and inadequate support and guidance, may prevent older people from using digital health services [15–17].

*Migrants,* with varying reasons for entering the country, such as family reasons, work, or refugee status, constitute an increasing group that can experience challenges in using digital health services in their new home country. Disparities have indeed been identified in digital health and in migrants’ access to digital health services in different societal contexts [18–20]. Previous studies have found, for example, that migrants and ethnic minorities search for health information online less often than the general population [6, 21, 22]. Migrant background has also been associated with lower understanding of the web-based health information [23].

The *‘high users’ of health services* often have a chronic disease or disability requiring regular health check-ups and care. This group can be at a risk for digital exclusion as poorer health has been associated with lower levels of interest in digital health [24] and perceived benefits of its use [25]. Additionally, the evidence suggests that health systems do not sufficiently offer digital health for their complex health needs [26].

Another group whose well-being and continuity of care has been particularly at stake during the COVID-19 pandemic due to the avoidance of physical contact is *mental health service users* [27]. Poor mental health has been associated with negative attitudes towards digital health services, posing mental health service users, or those in the need of mental health services, at risk of digital exclusion [28]. Those with more severe mental disorders may also experience cognitive impairments that hamper the use of digital services and perpetuate digital exclusion alongside lack of digital skills or financial resources [29, 30].

*The unemployed* represent another group that is under the threat of digital exclusion. For example, Helsper and Reisdorf [13] have discovered, that the unemployed is one group that is likely to include Internet non-users. Reasons for this include lack of access, skills and financial resources but also motivational reasons such as lack of interest in the Internet. A Finnish study discovered that unemployed persons are lacking skills for using digital health services and use digital services less often than others [31].

### The present study

Obtaining up-to-date information on the experiences of vulnerable groups about the challenges related to the use of digital health services is paramount because the perspective of these groups has not yet been adequately addressed during the COVID-19 pandemic [5]. This task is important even if the COVID-crisis subsides, since the effects of the crisis on health are likely to be felt long after the pandemic. It is also likely that the provision of digital services will continue to grow alongside or instead of the traditional face-to-face services. While those at risk of digital exclusion are not a homogeneous group, qualitative interviews exploring the experiences of different groups and communities and thereby actively involving them is essential for the development of future digital services.

The aim of this qualitative study is to examine the challenges experienced by vulnerable groups in using digital health services during the COVID-19 pandemic.

The identified elements are viewed and synthesized by utilizing the DHEF framework [11].

## Methods

### Context

In Finland, universal access and patients’ equal rights to public health services are defined by law [32, 33]. In 2019, Finland also enacted a law on the provision of digital services, which aims to promote their availability, quality, information security and equal opportunities for people to use digital services [34]. Although Finland is a high-income country with advanced health policy programs, there are health inequalities related to socio-economic status. This is reflected in higher levels of morbidity and mortality in vulnerable groups, who often have cumulative social disadvantages, such as low levels of education, low income, and unemployment [35, 36].

Public health services are divided into primary health care and special medical care, of which primary health care refers to health center services organized by municipalities, including the promotion of the well-being and health of the population and the prevention, diagnosis and treatment of diseases. In addition, health services are provided by private service providers including occupational health care. The third sector complements the provision. Their main tasks include the organization and implementation of volunteering and assistance work, as well as the provision of health services and related development activities complementing public services. Due to their typically having low income, vulnerable groups tend to rely on public health services and the third sector.

Finland is one of the forerunners of digitalization [37]. However, according to Statistics Finland, in 2019 up to 10% of the population did not have a computer at home, 17% did not own a smartphone and 8% did not have access to the Internet. Admittedly, in 2020 a considerable increase was seen in the use of the Internet for social networking and communication in Finland, especially in the oldest age groups [38], most likely due to COVID-19 crisis and the restrictions that forced social interaction online, pushing people to learn new social media skills. However, the ability to use the Internet does not necessarily imply an ability to use digital health services: a recent population survey (conducted during the COVID-19 pandemic) showed that only 22% of Finnish adults had communicated digitally with a social or health care professional [39].

This study was carried out as part of the DigiIN research project, funded by the Strategic Research Council, which aims to create solutions which will ensure that the social welfare and healthcare sector’s digital services are available and accessible to everyone. The authors are researchers of the project and are not involved in the provision or actual development of digital health services.

### Study design

The research design was a descriptive qualitative study based on semi-structured individual interviews among five vulnerable groups at risk for digital exclusion.

### Recruitment of participants

The recruitment process varied by group (detailed information is provided in Table S1 in Additional file 1). Participants were mainly recruited through a convenience sampling from third sector organizations that provided services for the target groups across Finland. With the help of the organizations, we forwarded an invitation letter to their clients, and those interested contacted the researcher, or the organizations delivered the client’s contact information to the researcher with the client’s permission. Some participants were reached through an online event or social media channels of the organizations, Facebook groups aimed at target groups, or using snowball sampling. As an exception, we reached high users by random sampling (*n* = 100) from the register data of one Finnish municipality. We mailed an invitation letter to these randomly selected persons, after which the researcher called everyone and inquired about their interest in participating. For participation in all groups, a small thank-you gift and an opportunity to participate in a lottery on a tablet computer were offered.

The participants (*N* = 74) were older adults (*n* = 16), older Russian-speaking migrants (*n* = 6), mental health service users (*n* = 12), high users of health services (*n* = 17), the unemployed (n = 16), and Russian-speaking unemployed migrants (*n* = 7). Russian speakers are the largest migrant group in Finland [40]. Most participants in the groups were women, except high users included more men. On average, the oldest participants (mean 75.4 years) were in the group of older people while mental health service users were on average the youngest (mean 30.7 years). Mental health service users, older migrants, and unemployed migrants had typically a higher education degree while the others most often had a secondary education. The life situations were heterogeneous within and between groups. Detailed information about the participants can be viewed in Table S2 in Additional file 1.

### Data collection

Data were collected using a semi-structured interview guide (Text file S1 in Additional file 1), jointly produced by the research group. The participants were told about the definition of digital services with concrete examples in the context of the Finnish health services. We asked questions such as what kind of experiences participants had about digital services during the pandemic, whether they perceived benefits from digital services or possible reasons for not benefiting, and how they considered that digital services could be developed. We piloted the interview guide with one older person and three mental health service users. No major changes were required to the interview guide, so the pilot interviews were included in the study with the permission of the participants.

Individual interviews were conducted by phone between October 2020 and May 2021 (the second and third waves of the COVID-19 pandemic in Finland). Each group of the participants had their own interviewer. The other groups were interviewed in Finnish but the migrants were interviewed in their native language Russian. The interviews were recorded into digital audio recordings with the permission of the participants. The mean length was 39 min (range 16–90 min) with the longest on average in older adults and shortest in high users. Transcription companies transcribed the recordings, resulting in a total of 1044 pages of transcribed text (Times New Roman font size 12, line spacing 1.5). The Russian transcripts were translated into Finnish for analysis.

### Data analysis

We applied inductive and deductive content analysis to analyze the qualitative data [41]. The combined approach allowed us to first identify emerging themes in the data and then synthesize the findings using an existing framework [11] to develop a deeper understanding of the phenomenon.

The data analysis included three iterative steps of data reduction, grouping, and abstraction [41]. The unit of analysis was a set of ideas in which the participant described challenges related to digital health services. In the data reduction, researchers (LV, PV, LH, JK, UB) read through the transcripts and used open coding to summarize the set of ideas as accurately as possible. Each researcher coded the interview transcripts of one vulnerable group, whose interviews they had also conducted. Except for the migrants, whose interviews were conducted by one researcher in Russian, and another researcher coded the data after it was translated into Finnish. Codes were then separately grouped within each client group based on similarity and given a descriptive name. These subcategories (*n* = 44) were then reviewed by one researcher (A-MK) who further categorized them into upper categories (*n* = 6) that were retrieved from the digital determinants of health of the DHEF [11]. Discussions were held with the research group about the categorization to reach a consensus.

In the Results section, we provide direct quotations from the interviews (translated from Finnish into English).

## Results

The elements related to digital determinants of health that challenged the opportunities of vulnerable groups to benefit from digital health services were partly congruent, but also unique. The group-specific challenges for each determinant are presented separately in Table 1 and results are summarized in the following sections.

**Table 1:** Challenges related to digital determinants of health experienced by different vulnerable groups

Digital determinant of health	Older adults	Migrants	Mental health service users	High users of health services	The unemployed
**(1) Access to digital resources**	Lack of basic computer skills Lack of suitable devices to use digital health services Lack of support and training to take advantage of digital health services Hearing disability reducing the capability to use remote option	Lack of strong electronic identification (e-ID) Insufficient local language skills to use digital health services Lack of specific digital skills required for digital health services.	Inadequate digital skills to take advantage of a variety of digital health services	Inadequate digital skills to access and use digital health services Insufficient English language skills challenging the Internet use Poor health complicates learning and using digital health services Learning to use digital health services requires too much effort	Usability issues challenge finding information on the websites of some health services Dysfunctional, too old or otherwise inappropriate devices hinder the possibilities of using digital health services Dysfunctional internet connections at home
**(2) Use of digital resources for health seeking**	Digital health services are not applicable for all health care needs	Handling more demanding and complex issues is poorly managed in a digital environment Risk of being misunderstood in a digital environment due to language issues	In the digital environment, interaction and communication are perceived poor Lack of private space complicates the use of digital health services		Lack of private space induces privacy issues and difficulties to concentrate on discussing with a health professional
**(3) Digital health literacy**					
**(4) Beliefs about potential of digital health to be helpful or harmful**	Fear of using and making mistakes in digital health services Distrust for the quality of remote health services	Security concerns and lack of trust in digital health platforms Fear of making mistakes that can have serious consequences	Security issues complicate the use of digital health services Lack of an incentive to go out of the house when using digital health services	Insufficient data security skills	
**(5) Values and cultural norms/preferences for use of digital resources**	Preferring face-to-face services when living next to a service provider Lack of interest to use computer or smartphones Hesitative attitude towards digital health services	In a digital environment, valued nonverbal and personal communication is lacking	Preferring face-to-face consultation because remote feels unusual	Digital health services are not seen to have added value Identifying the service needs remotely by a health care professional requires more time and patients’ effort “An old-school mind” preferring face-to-face services	Preferring face-to-face consultations because seeing a person’s face makes communication easier Lack of interest in using or learning how to use digital devices
**6) Integration of digital resources into community and health infrastructure**	Lack of awareness of available digital health services and their value	Lack of digital health services and websites in participants’ native language	Remote option is not always available in health services	Not being informed about a remote option for health services	Digital health consultations not always available Information transfer between different systems does not always function properly Not always possible to interact with the service provider

### Access to digital resources

For the most participants, access to digital health services was hampered by insufficient digital skills, language skills, or both. In addition, a lack of support and training, poor health, and the lack of strong e-identification or suitable devices required for digital services prevented the access.

Regardless of age, the participants felt that the use of digital health services required significantly higher-level digital skills compared to the skills required to use every day digital devices and applications.

Especially for many older participants, poor basic computer skills and the lack of devices were considerable barriers to access digital health services. However, there were large differences in skills, and some older participants were able to use computers and smart devices completely fluently.

*“These digital gadgets require a lot of competence, a lot of knowledge, skills that people in my age don’t naturally have. Such competence, know-how. That’s a bad thing. The terminology is unfamiliar, I don’t even understand the questions of what’s out there.”* (Older adult, 69 years)

Older participants felt that they did not receive enough guidance on how to take advantage of digital health services. In the past, voluntary support and computer assistance had helped many older adults to access digital services, but due to the COVID-19 pandemic, many of the trainings or digital support had been canceled or converted into online remote events. Participants experienced difficulties in finding or joining these remote support events or existing equipment did not allow participation.

Some high users also described that learning digital skills to access digital health services was too demanding and time- and energy-consuming. Simultaneously, they raised concerns that they might fall out of a digitalizing society because they felt that their current life situation did not allow for learning.

*Then I’ll fall out completely if I’m not there. It arouses fear and anxiety if you think about it. I’ve so much of everything else, I can’t get acquainted. I must manage to focus on and delve into it, then I’d learn those digital services. If someone were teaching nicely. But I should have enough strength to focus on the thing that I have enough strength to learn it. I should take time for that.* (High user 15, 55 years)

High users and mental health service users also described that they had ability to access certain health services but considered access challenging to others, mostly due to problems in usability or language. Some participants described that they could not access the digital services at all independently.

Inadequate local language skills were shown to be a major barrier to migrant participants’ use of digital health services. Even booking appointments remotely using the Finnish language proved difficult for some participants. Moreover, in Finland, to be able to use digital public services one must have a strong electronic identification (e-ID). Obtaining an e-ID is not straightforward to non-EU migrants.

*“In mastering language and computer, that’s my difficulty. My main challenge, yes, because you must use the language remotely, filling out the forms correctly, there you need to answer the questions, but you just don’t always succeed in it correctly. I use a translator for some things, translate something myself with the help of a dictionary but the translations aren’t always very accurate. There you’re afraid to make a mistake because it’s an official document.”* (Older migrant, 72 years)

The migrant participants also pointed out that the use of digital services does not only demand a high level of local language skills, but also require mastering of specific administrative and medical vocabulary. This experience was shared by Finland-born older adults and high users who thought that services can include too difficult language or professional vocabulary.

For high users, poor health seemed to particularly challenge the use of digital health services. For example, some explained that they had even had a career in IT, but memory impairment had complicated keeping up with the changes and learning. A language disorder was a perceived barrier to use remote health consultations because the expression of service needs was considered more difficult without physical presence. Visual impairment was also described to challenge on-screen reading, and therefore, interviewees had preferred obtaining health information in a letter or they had printed it from a digital service on paper for reading. For some older participants, having a hearing disability also reduced the desire to use phone as a remote option.

For some of the participants, such as the unemployed and older adults, the lack of suitable devices, due to financial reasons or problems with the devices’ functioning at home for example, hindered significantly their possibilities to use and benefit from digital health services. Because of these device issues, some of the participants had been forced to seek services elsewhere.

### The use of digital resources for health seeking

The participants highlighted that digital services were not applicable for all situations. Many of the participants experienced challenges related to the nature of communication and poor interaction in the digital environment.

Face-to-face services were perceived as a more effective in handling more demanding and complex matters, whereas, easy-to-do, or routine-like issues, such as booking appointments and checking the health records online were considered as more doable digitally. However, communicating was not always possible in services.

*” The thing that annoys me in the My Kanta pages [where you can see your own health records] is that they are not reciprocal. You cannot comment anything there even if you find a clear mistake in the text written by a physician. They could add some chat function somewhere in there to enable commenting.”* (Unemployed, 45 years)

Among migrant participants, the complexity of the health issue was also interconnected with local language skills. Face-to-face meetings were thought to reduce the misunderstandings because they provided the possibility to clarify the issue, ask questions, and also increased the experience that their health issue was properly understood. Similar experiences were expressed by high users and mental health services users, who felt that visiting the service physically would allow the professional to ask, see, and feel the situation comprehensively.

Poor interaction was considered as one of the major barriers to benefitting from digital services among the mental health service users. Some perceived it difficult to express their health service needs remotely. Mental health service users also felt that digital mental health consultations lacked warmth and the conversation felt distant without facial expressions and tones. Some described that in the remote group therapy sessions, people talked easily on top of each other, and therefore, the health professional had to assign turns to speak, which disturbed the natural rhythm of the conversation.

Some participants also described that using digital health services at home in the presence of another person (such as remote consultations or therapy sessions) was challenging and did not allow for privacy.

*“We had a situation where I was at home with our child. How can you focus on discussing when you have a toddler rolling around? We also have a small home, so we do not have a place where I could lock myself into so that nobody is listening.”* (Unemployed, 34 years)

### Digital health literacy

The present study used the definition of digital health literacy as the degree to which individuals have the capacity to obtain, process, and understand basic health information from electronic sources to make appropriate health decisions [42]. Based on this definition, digital health literacy was not an issue that participants would have raised as a challenge.

### Beliefs about potential of digital health to be helpful or harmful

Participants commonly raised some issues and concerns related to the security of digital health services, as well as fear and lack of trust regarding digital platforms.

Particularly due to the sensitivity of mental health issues, the mental health service users perceived that they did not feel as secure to discuss these matters remotely. The smaller group sizes meant that the service users felt safer in a digital environment as the interviewees described that the more customers were present, the less comfortable they were to discuss openly about their sensitive matters.

*“People might not dare to talk about such personal matters as much via a computer compared to when we would see physically. I’ve also experienced it, especially if there are several listeners. But maybe if there were only a few people online, then maybe I could dare to talk about such more personal things. If there are more, then no.“* (Mental health service user, 40 years)

Digital health platforms were not necessarily considered safe systems, which generated security concerns that the personal data might be compromised. The rapid transition from face-to-face meetings to digital service due to the COVID-19 pandemic in Finland had sometimes resulted in providing services (e.g, a service supporting mental health provided by third sector organization) on platforms that were perceived insecure. Moreover, lack of sufficient security expertise worried the participants, as they felt that they did not have the ability to protect their computer from hackers, for example. They acknowledged that their individual computer behavior might cause risks for their health data if they used health services digitally.

Fear of making mistakes while using digital health services emerged especially among the older participants and migrants. They mostly feared that when trying to use digital services, something irreversible would happen and everything would go wrong. They doubted whether they would get any support if something goes wrong with the digital service. Because mistakes were thought to lead to potential significant errors with long-term consequences, such as non-renewal of prescriptions, delays in service or treatment or compromising their health information, participants were worried about using digital services and therefore contact-based service options (especially face-to-face meetings) were favored.

*” When there’s no helper, if it’s the first time you’re using the system, you’ll face an obstacle. And there it’ll stymie that you’re afraid of pressing something wrong, that the computer crashes, the programs crash. Such senility of old age. The programs are difficult for us to use.”* (Older migrant, 64 years)

In addition to safety concerns, some mental health service users described digital health services as a potential harm for mental health as they perceived that compared to attending traditional face-to face appointments, digital services lack an incentive to leave the house. The older participants similarly pointed out that digital services reduced their opportunities for physical activity, which was important for their well-being.

Experiences of distrust for the quality of digital health also emerged from the interviews and some participants indicated having less confidence in digital health services than in the face-to-face services.

### Values and cultural norms/preferences for use of digital resources

Interviewees widely indicated their preference for traditional face-to-face meetings over digital ones, as digital services were not seen to provide a service experience equivalent to a face-to-face encounter. Many did not see the added value of digital services.

Among the migrant participants, the preference for face-to-face appointments was strong because they valued personal communication, including gestures, facial expressions, and touches. They felt that possibilities of showing emotions and using non-verbal communication disappear in digital services. Additionally, the contact with a health care professional was considered less personalized in digital health services compared to traditional face-to-face services.

*“Personally, I like to visit in person because this human factor is very important to me as a computer is a computer, but when you talk to a person face-to-face, it’s a whole different thing.”* (Unemployed migrant, 49 years)

Some of the high users perceived that identifying the service needs remotely requires more time and patients’ investment compared to face-to-face services, and therefore, they preferred visiting health services physically. Some older adults also felt that remote health service options, such as phone calls, were inconvenient as they often required time and waiting in line.

*“Preferably I’d go physically to the service. There they’ll check the situation comprehensively. That I can say all things out. It’s possible even remotely, but it takes more time to discuss everything through calmly.* (High user, 55 years)

Some older adults had doubts about treating health issues remotely, and things were thought to be handled worse or not at all compared to face-to-face services. In this group, lack of interest to use a computer or smartphone was also common. High users and the unemployed shared their thoughts and feelings about digital health services which tended to be negative irrespective of their level of digital skills. The participants often explained their attitudes by having an “old-school mind” and their habit of using health services physically.

Additionally, digital health services were not seen to have an added value when the participants repeated the view that living close to a health care center or a visit by a health care professional in a sheltered housing provided an easy access to care and created no need to use health consultations remotely.

### Integration of digital resources into community and health infrastructure

The challenges associated with integrating digital resources into health infrastructure were mainly related to either the fact that digital alternatives were not always available as desired by participants, or participants were unaware of existing digital service options and their value.

In particular, some of the high users hoped that service providers would better inform them about the possibilities to use digital health services. Despite the high use of health services, some had never been instructed to take advantage of different digital platforms such as electronic health records. Moreover, it seems that in some cases, there was actually no option provided to a patient to use a health service remotely at all. Indeed, some of the mental health service users and the unemployed wondered why the public health sector did not provide more options to use services remotely, such as making appointments online or having health consultations via video call.

Among the migrant participants, one key challenge that emerged was that only some of the webpages were available in the participants’ mother tongue, Russian, despite Russian being the most widely spoken native language in Finland after Finnish and Swedish. Relevant information was difficult to find on the websites and the information was provided in a difficult to understand, not intuitive manner.

## Discussion

In this study we investigated the challenges experienced by vulnerable groups in using digital health services during the COVID-19 pandemic. Given the significant emphasis on the role of digital health services during the pandemic, which may also have increased health inequalities [2, 5, 6], this study provides valuable up-to-date information on those groups at particular risk of digital exclusion.

### Principal findings and comparison with prior work

The results of this study indicate that vulnerable groups experienced problems with many digital determinants of health. The most obvious barriers to using the digital health services were the various challenges related to access to digital resources. The most typical challenge was that individuals, regardless of the age or group, felt that their skills were insufficient to access the services. Previous studies have also identified that inadequate digital skills widely affect vulnerable groups and the problem is not limited to older adults [26, 43, 44]. In contrast to previous studies, the notable finding of this study was that individuals may have good digital skills as such, and they may use the Internet and social media several times a day, but these skills did not seem to guarantee the ability to cope with the use of digital health services. This was mostly due to poor usability, difficult vocabulary in the services, or weak local language skills. This finding is in line with previous findings showing these factors as significant barriers to adoption of digital health services among different vulnerable groups [22, 23, 43, 45–47]. In addition to the previous studies showing that migrants struggle with the difficult-to-understand language of the services, our results show that this challenge also affected those who spoke native language.

Weak digital skills were often linked to another common problem in access to digital resources: experiencing inadequate and difficult-to-find support for service use. Although this need for support and technical assistance has been discussed for years [48, 49], it appears that despite the constant increase in digital services, support is still not adequate or appropriate for different users.

Not having an e-ID was a considerable barrier for some migrant participants. In Finland, as in other Nordic countries, having an e-identification is a prerequisite for using digital health services and the e-ID system relies mainly on online-banking identification methods [50, 51]. To be able to authenticate in digital services, migrants from outside the EU must apply for a Finnish ID card first, and then apply for the possibility to have e-ID linked to their banking identifiers. This authentication problem has been noted previously, as in 2018, 98% of the general adult population in Finland were reported to have e-ID, while only 88% of migrant adults had it [52].

Participants commonly experienced that communication-related weaknesses prevented their use of digital resources for health care seeking. Due to poor communication, digital health services were not yet perceived to be able to meet complex service needs or the health issue were not seen to be fully addressed at once. Previous research has found similar kind of results showing that lack of interaction poses challenges to digitalization especially in mental health services, where patient-professional dialogue and interaction play a key role [53–55]. However, our findings add to these indicating that communication challenges in digital services can affect various service users and the challenges experienced also vary for different user groups.

An increased use of digital health services is known to increase the risk of security and privacy vulnerabilities [56]. This seemed to be a concern also in this study as the most typical belief about the potential harm of digital health. For many, fear and lack of trust hampered the use. This is in line with previous studies stating that security, privacy, and confidentiality concerns are considerable barriers to accessing digital health services for various vulnerable groups, such as mental health service users, people with multimorbidity, or older adults [24, 26, 49, 53, 55]. However, our results also emerged that the safe use of digital services require external resources for the private and confidential environment in which the service is used. We showed that privacy and confidentiality issues can be especially visible for those who are unable to use digital health services in private place or independently when sensitive information may need to be shared with another.

This study showed that traditional face-to-face health services are often valued and preferred over digital services. Some participants admitted to having a low motivation towards digital solutions, which is why the threshold for using digital services was partly high. The importance of attitudes and motivation in benefiting from digital services has been highlighted in previous studies as well [13, 47, 57]. Participants’ attitudes may have been influenced by the fact that many of them did not know that digital services were available and worth using. Thus, it seems that knowledge of digital services and their potential to promote health and well-being does not yet seem to reach everyone.

### Practical implications: areas for the development of digital health services

**Fig. 1:**
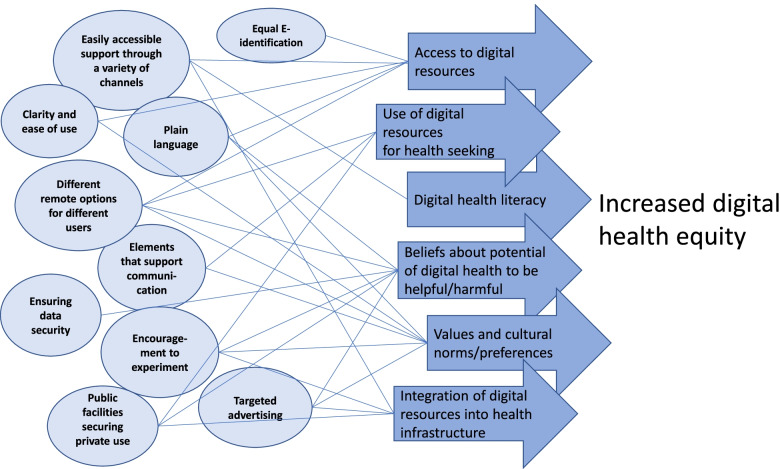
Suggestions for development to improve the accessibility of digital health services and increase digital health equity

Mitigating strategies such as increasing physical access, digital skills and social support and improving the digital remote support infrastructures have previously been proposed to reduce digital inequalities and increase the use of technology and digital services [5, 6]. Moreover, the importance of hybrid strategies, including both high- and low-tech perspective and combination of online and offline strategies has been highlighted [2]. Based on the identified challenges experienced by vulnerable groups, we are able to suggest several areas for development that could improve the more equitable accessibility of digital health services. Development must focus both on better usability of digital services and on the opportunities and ability of individuals to benefit from them. Figure 1 illustrates the areas for development, which are described in more detail in Additional file 2.

### Strengths and limitations

The main strength of this study is the large and diverse group of participants who are at a particular risk of being excluded from digital health services. The data collected during the COVID-19 pandemic also provide very up-to-date and unique information on the experiences of these vulnerable groups at this exceptional time. The fact that we did not directly ask the participants about the challenges related to certain digital determinants of health, but instead gave them a more open opportunity to raise the most important challenges for themselves, may have affected the content of the results. For example, it is possible that if we had asked directly about the challenges related to digital health literacy, the participants could have described them. Although in this study we looked at the participants’ experiences as part of the vulnerable group in which they were recruited, in practice the challenges related to digital services also varied within the groups. This shows that there are no convergent vulnerable groups, but individuals with individual needs and challenges.

We did not conduct a ‘member checking’ and ask participants to check the accuracy of the results in relation to their experiences, which would have increased the credibility of the results. However, the key strength of this study is researcher triangulation, as several researchers participated in the implementation of the study and in the analysis and interpretation of the data and findings. This allowed for a broad examination of the phenomenon and strengthens the validity of the results [58].

## Conclusions

This study reinforces the view that not all people have been equally able to access and benefit from digital health services during the COVID-19 pandemic. Our results suggest that the major problems in accessing digital health services seem to be related to individuals’ access to digital resources, although, this study identified significant challenges in other digital determinants as well. There are several reasons for digital exclusion, such as insufficient digital skills or local language skills, or the fact that the use of digital services requires such effort and resources, which may be too much for some in their life situation. The health care needs of vulnerable people may also be complex and digital health services do not yet appear to be fully responsive to needs that require close interaction or clarification. In the future, it will be important to invest in information about digital health services through various channels because the opportunities and potential benefits of these services has not been disseminated widely enough to reach everyone. Moreover, traditional face-to-face health services will continue to be important and should still be maintained and provided alongside digital health services as there will always be people (eg, older adults with cognitive decline or sensory impairments, and illiterate migrants) who are not able to use digital health services.

## Supplementary Information

**Additional file 1: Table S1.** The eligibility and recruitment process of the participants. **Table S2.** The demographics and description of the study participants. **Text file S1.** The interview guide.

**Additional file 2.** Descriptions on the suggestions for development to improve the accessibility of digital health services and increase digital health equity.

## Data Availability

The datasets generated and analysed during the current study are not publicly available for the protection of the anonymity of the participants but are available from the corresponding author on reasonable request.
