# Supplementary material for: Towards digital health equity - a qualitative study of the challenges experienced by vulnerable groups in using digital health services in the COVID-19 era
Source: BMC Health Serv Res. 2022 Feb 12;22:188. doi: 10.1186/s12913-022-07584-4 (PMC8840681; doi:10.1186/s12913-022-07584-4)
Supplement: Supplementary file 1 — Additional file 1: Table S1. The eligibility and recruitment process of the participants. Table S2. The demographics and description of the study participants. Text file S1. The interview guide. [file 12913_2022_7584_MOESM1_ESM.docx]

Additional file 1

Table S1. The eligibility and recruitment process of the participants.

| Vulnerable group | Eligibility criteria | Sampling method | Recruitment location | Method of approach |
| --- | --- | --- | --- | --- |
|  |  |  |  |  |
| Older adults | Approx. 65–90-year-old people in different life situations | Convenience sampling, snowball sampling | Third sector organizations (n=2) which, based on volunteering, provided peer support and digital guidance for older adults or promoted the issues of the older adults across Finland. | 1) An information letter about the research and eligibility criteria were delivered to the organizations who provided contact details of potential participants with the permission of the client. The researcher contacted the potential participants and presented the study, after which the potential participants decided whether to participate.  2) The researcher asked the participants whether they could promote the study to other older adults they know. |
| Migrants |  |  |  |  |
|  | Unemployed Russian-speaking migrants who were unemployed and permanently residing in Finland | Convenience sampling | A third sector organization which hosts events for unemployed Russian-speaking migrants and Facebook groups targeting Russian-speaking migrants | 1) The researcher went physically to the place where the organization arranged workshops and asked potential participants to participate.  2) The study was promoted on Facebook groups and interested participants contacted the researcher. |
|  | Older Russian-speaking migrants aged 64 or older permanently residing in Finland | Convenience sampling | A third sector organization that promotes multiculturalism and well-being | The organization contacted potential participants and provided researchers with the contact details of those interested in the study with the permission of the participants. |
| Mental health service users | Adults who experienced any symptom and severity of mental disorder and used mental health services before or during the pandemic, or both | Convenience sampling, purposive sampling | Third sector organizations (n=6) that provided support groups and day activities to promote mental health, well-being, social inclusion and work and functional capabilities across Finland | 1) The organizations forwarded an invitation letter by e-mail to their clients, and the interested participants contacted the researcher.  2) The organizations identified eligible participants who could be informative for the study among their clients and asked face-to-face or remotely for their interest in participating. The interested participants asked the organizations to deliver their contact details to the researcher.  3) The researcher presented the research on a digital event of one organization and scheduled interviews with interested participants through private messages. |
| High users of health services | Adults who had used outpatient health services at least eight times per year for 3 of the 4 follow-up years between 2017 and 2020 and had a health condition allowing an independent participation | Random sampling | Register data (n=100) of a Finnish municipality | The names, mail addresses, and phone numbers were retrieved from the register data. An invitation letter was mailed, after which a researcher called everyone and inquired about their interest in participating in the study. |
| Unemployed | Adults who were long-term unemployed or unemployed during the pandemic. Some were engaged in employment-promoting services such as a rehabilitative or voluntary work | Convenience sampling | Third sector organizations (n=3) that provided a meeting point for people in a vulnerable position, employment-promoting training or rehabilitative work services, and Facebook groups targeting unemployed people | 1) The organizations disseminated an invitation letter on their web page, social media channels, and in some cases, further to the professionals working with unemployed clients. The interested participants contacted the interviewer or asked the professionals to deliver their contact details to the interviewer.  2) The research call was posted on the Facebook groups targeting unemployed people and interested participants contacted the researcher. |

Table S2. The demographics and description of the study participants.

| Vulnerable group | | n | Age (years), mean (range) | Gender, n | | | Typical educational level | Description |
| --- | --- | --- | --- | --- | --- | --- | --- | --- |
|  |  |  |  | Woman | Man | Non-binary |  |  |
|  | |  |  |  |  |  |  |  |
| Older adults | | 16 | 75.4 (67–90) | 10 | 6 |  | Secondary edu-cation | Lived in diverse residences. |
| Migrants | |  |  |  |  |  |  |  |
|  | Unemployed Russian-speaking migrants | 7 | 39.9 (35–49) | 5 | 2 |  | Degree | Had migrated from Russia or other ex-Soviet countries to Finland several years ago and became unemployed before or during the pandemic. |
|  | Older  Russian-speaking migrants | 6 | 72.5 (64–82) | 5 | 1 |  | Degree | Had migrated from Russia or other ex-Soviet countries and lived for a long time in Finland. |
| Mental health service users | | 12 | 30.7 (23–46) | 8 | 4 | 1 | Degree | Experienced different symptoms and severity of mental health conditions. Half were students or employed, others half unemployed, in rehabilitative work, or laid off during the pandemic. |
| High users of health services | | 17 | 64.1 (53–79) | 5 | 12 |  | Secondary edu-cation | Some had a stable health condition and were able to function in working life, others had complex multi-morbidities or disabilities and were on sickness pension or old-age pension. |
| Unemployed | | 16 | 50.0 (31–63) | 9 | 7 |  | Secondary edu-cation | Some had been unemployed for a long-term, others were in rehabilitative work, volunteering, or part-time work. |

Text file S1. The interview guide

1. Warm-up: How would you describe your life situation before the corona pandemic? What health and social services did you use before the corona pandemic?
2. Have you used health services during the corona pandemic?
   1. Have the health services you used before corona been replaced by remote services? Have you used them? Why or why not? (If no: Have you been left without health services because they have not been provided physically?)
   2. Have most of the health services you have used during corona been provided physically or through remote connections?
3. During the corona pandemic, have you used other services, such as social services?
   1. Have the services you used before corona been replaced by remote services? Have you used them? Why or why not? (If no: Have you been left without services because they have not been provided physically?)
   2. Have most of the services you have used during corona been provided physically or through remote connections?
4. Could you please share your experiences of using remote health and social services during the corona pandemic?
   1. In your opinion, have the use of remote services given you help, support or other benefits? What kind of? (If no: Why?)
   2. How do you think remote services have functioned? What has functioned well? What has not functioned well?
   3. How do you think remote services should be developed?
5. How has the corona pandemic affected your social relationships?
   1. Have you experienced loneliness during the corona pandemic?
   2. Have you used a smartphone, computer/laptop or tablet to communicate with others during corona? What device have you used? What software or platform have you used?
   3. Do you feel that digital communication has affected your experience of loneliness? If so, which device and software/platform? How have they affected your experience of loneliness?
   4. How could loneliness be relieved using information technology?
6. How does digitalization influence your life now during the corona pandemic?
   1. What thoughts and feelings have emerged for you? Could you give some examples?
7. Background questions: Age, gender, and country of birth? How many years in total have you had education?
