# Supplementary material for: Towards digital health equity - a qualitative study of the challenges experienced by vulnerable groups in using digital health services in the COVID-19 era
Source: BMC Health Serv Res. 2022 Feb 12;22:188. doi: 10.1186/s12913-022-07584-4 (PMC8840681; doi:10.1186/s12913-022-07584-4)
Supplement: Supplementary file 2 — Additional file 2. Descriptions on the suggestions for development to improve the accessibility of digital health services and increase digital health equity. [file 12913_2022_7584_MOESM2_ESM.docx]

#### Additional file 2.

#### Descriptions on the suggestions for development to improve the accessibility of digital health services and increase digital health equity

#### Easily accessible support through a variety of channels

#### To be able to take full or even partial advantage of digital health services to manage their health, vulnerable people should be provided with more digital support and training. Public service providers should provide formal assistance and technical support with their digital service systems, in multiple languages. Instruction videos and remote support by using video connection could be provided and users could also benefit from demo versions of the services where they could test their use and functionality without fear of errors and their consequences. Overall, support should be easy to find, quickly available, and should be provided through channels and means familiar to different target groups. The accessibility of support and training should also be promoted by increasing information on them through channels that reach different users.

#### Clarity and ease of use of digital health services

Digital health services should be easier to use and more self-explanatory. Users of different skill levels should have the opportunity to access health services independently and would not always need to seek outside help when dealing with personal health issues. To achieve this, user interfaces should be logical, simple, and well designed to support end-user action and goal. Structuring of the information should be also improved, and search functions should be made more effective, so that needed information could be easily found and searched on the webpage by as many diverse users as possible.

#### Plain language of digital health services

Digital health services should be provided with user-friendly language. User interfaces and descriptions should be in plain language and complex or medical vocabulary should be avoided*.* The webpages of the digital health services should have information in easy language and have more language versions, including not only the official languages ​​of the country but also the other most commonly spoken "foreign" languages in the country.

#### Public facilities securing the private use of digital health services

Public institutions such as public libraries and educational institutions should build well-soundproofed and gaze-protected facilities that secure private use of digital health services. Health issues are sensitive and the provision of this type of facilities could particularly benefit individuals who do not privacy at home. Additionally, the facilities could overcome the lack of incentive to go outdoors experienced by the mental health service users and yet provide benefits over traditional physical services. These facilities should be easily available and close to residents.

#### Ensuring and monitoring the data security of services

The data security of different digital services must be uniformly guaranteed, constantly monitored and communicated to users. Ease of access to the service should not be achieved at the expense of data security. The security expertise of health care professionals and clients should also be ensured.

*Adding elements that support remote communication*

Certain elements could be developed to foster remote communication. Increasing the opportunities to use video calls in health services could better correspond to physical encounters by allowing the recognition of nonverbal gestures. Similarly, the development of digital elements for platforms, such as functionalities that facilitate the expression of emotional states, could bring new opportunities for interaction. In group-based mental health care, reducing group sizes could improve trust-building and open discussions between group members and the professionals. Healthcare professionals’ skills to work in digital environment with vulnerable groups should also be developed to reduce the sense of distance.

#### Encouragement to experiment with digital health services

The vulnerable groups need encouragement to experiment with digital access to health services. For that, the benefits of digital health services should be communicated in a clear and inspiring manner, preferably, personally through a trusted person, such as a familiar healthcare professional. Providing demo versions could also encourage users to experiment with the services.

#### Targeted advertising about digital health services

Digital health services should be effectively advertised to vulnerable groups to make them aware of the possibility of using the services remotely. Multiple communication channels are needed for information to reach different groups, for example, during a physical visit of the health service or through television, newspaper, and social media advertising.

*Different remote options to serve different groups and individuals*

Individual preferences or possibilities to use different remote options can vary so efforts should be made to diversify their provision. Some could benefit of the possibility to choose between video, audio, or chat health services while others can prefer a service without a real-time contact. However, the latter establish particularly high demands on, for example, usability, service language, and digital health literacy that should be considered.

#### Enabling equal E-identification

If a migrant fails to obtain an e-ID, they cannot access digital health services. Thus, the provision of public digital health services must ensure that everyone has the right to an easy-to-use, easily accessible and affordable e-ID.
